# Editorial: Dealing with unusual hosts and unconventional habitats: versatile strategies of *Salmonella enterica*

**DOI:** 10.3389/fmicb.2023.1342139

**Published:** 2023-12-18

**Authors:** Serena Ammendola, Shirley A. Micallef, Adam Schikora

**Affiliations:** ^1^Department of Biology, Tor Vergata University of Rome, Rome, Italy; ^2^Department of Plant Science and Landscape Architecture, University of Maryland, College Park, MD, United States; ^3^Center for Produce Safety and Security Systems, University of Maryland, College Park, MD, United States; ^4^Federal Research Centre for Cultivated Plants, Julius Kühn Institute, Institute for Epidemiology and Pathogen Diagnostics, Braunschweig, Germany

**Keywords:** *Salmonella*, unusual hosts, survival strategies, environmental reservoirs, *Salmonella*-plant interaction

*Salmonella enterica*, a member of the *Enterobacteriaceae* family, may cause gastroenteritis, typhoid fever, and systemic infections in a wide range of hosts, including humans, animals, and birds. Recent investigations revealed that *S. enterica* is a highly adaptable bacterium, capable of colonizing and persisting in unconventional habitats and hosts. This notion challenges the traditional understanding of *S. enterica* ecology and classical host-pathogen interaction studies.

A remarkable feature of *S. enterica* is its ability to adapt to diverse ecological niches. While this bacterium has long been associated with human and animal diseases, many studies have identified non-traditional hosts, such as plants, protozoa, and insects, as reservoirs for *S. enterica*. Understanding the mechanisms behind the adaptation to such a broad host range is crucial for comprehending the epidemiology and transmission dynamics of *Salmonella*-associated diseases. *Salmonella* has been found in water sources, soil, and even within biofilms on plastic surfaces. This versatility raises concerns regarding traditional sanitation and hygiene practices since *S. enterica* presence complicates efforts to prevent infections. Exploring the molecular mechanisms that allow *S. enterica* to persist in unusual environments is essential for developing effective strategies to mitigate contamination.

*Salmonella enterica* employs an array of survival strategies to thrive in unconventional hosts and habitats. These strategies include biofilm formation, stress response mechanisms, alternative metabolic pathways and the ability to change its phenotype. Biofilm formation enables *Salmonella* to adhere to surfaces, while stress responses enhance its resilience in challenging conditions. The understanding of *S. enterica* versatility is crucial for public health, because non-traditional hosts and unconventional habitats multiply the potential sources of infection. Furthermore, the ability of *Salmonella* to persist in diverse environments necessitates a holistic approach in order to prevent infections, involving not only traditional clinical interventions but also strategies targeting the environmental reservoirs.

The study by Han et al. explores *S. enterica* adaptation to agricultural environments. These environments, including soil and crop plants, serve as ecological niches for *Salmonella* and act as vectors for its transmission to the consumer. This study specifically investigated the adaptation strategy of *S. enterica* serovar Typhimurium, analyzing glycolysis and the tricarboxylic acid pathway intermediates in diverse agricultural settings. The authors identified several crucial genes, such as *aceE* and *aceB*, associated with *Salmonella* Typhimurium persistence in root or leaf tissues. *In vivo* persistence assays in tomato leaves further confirmed the significance of these genes in allowing *Salmonella* Typhimurium to adapt to agricultural environments. Additionally, the researchers unveiled a compensatory mechanism involving fumarate accumulation in response to mutations in a specific gene, *aceB*. These findings provide valuable insights into the complex mechanisms employed by *Salmonella* Typhimurium to regulate its metabolism in response to the diverse carbon sources present in agricultural habitats. Understanding these adaptation strategies seems crucial for developing effective strategies to reduce *Salmonella* persistence in food production systems, contributing to improved food safety.

The versatility of *Salmonella* Typhimurium in the colonization of different ecological niches depends also on its ability to respond efficiently to the fluctuation in micronutrient availability. Among these, zinc (Zn) plays an essential role in bacterial physiology. Although it is well-established that animals employ Zn nutritional immunity strategies to fend off pathogens, it is unknown if Zn is involved in *Salmonella*-plant interactions. In the study by Visconti et al., authors investigated the involvement of *Salmonella* Typhimurium Zn/Cd export systems in plant colonization using *Arabidopsis thaliana* as the model host. The authors demonstrated that *Salmonella* persistence in plant tissues was influenced by the Zn content and that, above a certain concentration, *Salmonella* expression of metal efflux pumps ZntA and ZitB was required for its survival in plants. The study intriguingly observed that the bacterial advantage in Zn detoxification becomes more pronounced in plant colonization under elevated Zn availability. Furthermore, this study revealed a fascinating aspect: the bacterial disadvantage associated with impaired Zn detoxification can be mitigated if the plant fails to efficiently translocate Zn to the shoots. This highlighted the intricate balance in *Salmonella*-plant interactions, where both bacterial adaptation strategies and host ability to transport Zn influenced the outcome. Importantly, the study proposed a potential strategy to control pathogen colonization via biofortification: namely by modulating plant metal content. Manipulation of the Zn level in plants could be an effective means of inhibiting *Salmonella* colonization.

In the context of food safety, the study by Liao et al. presents a promising approach to address the challenge of seed contamination with foodborne pathogens, specifically mung bean seeds. The authors explored the biocontrol potential of *Escherichia* phage Sa157lw. The significance of this research lies in its innovative strategy to combat contamination with pathogenic *Escherichia coli* and various *Salmonella* serovars in sprout production, where susceptibility to antimicrobials and the escalating issue of antimicrobial resistance pose urgent concerns. The detailed characterization of a phage (Sa157lw), including whole-genome sequencing and biological assessments, provides a robust foundation for understanding its biocontrol potential. The application of Sa157lw on mung bean seeds demonstrated significant antimicrobial effects, as evidenced by the substantial reductions in both *E. coli* O157:H7 and *Salmonella* Typhimurium after storage. The research offers an innovative antimicrobial intervention, addressing the pressing need for effective strategies in the face of increasing antimicrobial resistance (AMR) and susceptibility challenges in sprout production.

The study by Guan et al. provides a comprehensive exploration of *S. enterica* in an unconventional habitat, namely waterfowl, shedding light on the dynamic nature of AMR and the potential public health risks associated with these avian hosts. The research, conducted in Sichuan, China, emphasized the scarcity of systematic studies on *Salmonella* prevalence in waterfowl species, despite their significance as major reservoirs and sources for *Salmonella* transmission. The recovery and characterization of Salmonella isolates from two distinct collection periods revealed a diversity of serovars harboring multidrug resistance patterns. Whole-genome sequencing unveiled a complex array of AMR genes, including efflux pump genes, specific resistance genes, and notable temporal variations in resistance patterns. The identification of resistance genes like *tet(A)/tet(B)* and *catII* in the first collection period and *gyrA/gyrB* mutations in the second period pointed to shifts in AMR genes over time. Moreover, the detection of incompatible plasmid replicon fragments highlighted the potential for horizontal transmission of AMR genes, contributing even further to the competitive advantage of *Salmonella* in highly diverse microbial habitats. This insight into the genetic diversity and adaptive mechanisms of *Salmonella* in waterfowl populations contributes crucial information for understanding the evolution of AMR and the associated risks to public health.

The genetic diversity of *Salmonella* isolates, collected in Shenzhen, China over an 11-year period of time, was analyzed in the study by Luo et al.. The research, using whole-genome sequencing and drug resistance phenotyping, explored population dynamics, inter-host relationships, drug resistance, and food-related transmission risks. The identification of two main sequence types, that are becoming increasingly prevalent in recent years, underscored the dynamic nature of *Salmonella* genetic diversity and its potential impact on public health.

In conclusion, the ability of *Salmonella* to thrive in unusual hosts and unconventional habitats highlights its remarkable adaptability. Exploring the molecular mechanisms and the genetic diversity behind this versatility is essential for developing effective preventive and control measures and predictive models. Therefore, our increasing understanding of *Salmonella* ecology renders the consideration of a broader spectrum of hosts and habitats essential, in order to comprehensively address the challenges posed by this pathogen to public health and microbial ecology.

## Author contributions

SA: Writing – original draft, Writing – review & editing. SM: Writing – review & editing. AS: Writing – review & editing.

